# Development of amyloid beta gold nanorod aggregates as optoacoustic probes

**DOI:** 10.1371/journal.pone.0259608

**Published:** 2022-03-25

**Authors:** Mahmoud G. Soliman, Hannah A. Davies, Jack Sharkey, Raphaël Lévy, Jillian Madine

**Affiliations:** 1 Institute of Systems, Molecular and Integrative Biology, University of Liverpool, Liverpool, United Kingdom; 2 Physics Department, Faculty of Science, Al-Azhar University, Cairo, Egypt; 3 Centre for Pre-Clinical Imaging, University of Liverpool, Liverpool, United Kingdom; 4 Université Sorbonne Paris Nord and Université de Paris, INSERM, LVTS, Paris, France; Academica Sinica, TAIWAN

## Abstract

Propagation of small amyloid beta (Aβ) aggregates (or seeds) has been suggested as a potential mechanism of Alzheimer’s disease progression. Monitoring the propagation of Aβ seeds in an organism would enable testing of this hypothesis and, if confirmed, provide mechanistic insights. This requires a contrast agent for long-term tracking of the seeds. Gold nanorods combine several attractive features for this challenging task, in particular, their strong absorbance in the infrared (enabling optoacoustic imaging) and the availability of several established protocols for surface functionalisation. In this work, polymer-coated gold nanorods were conjugated with anti-Aβ antibodies and attached to pre-formed Aβ seeds. The resulting complexes were characterised for their optical properties by UV/Vis spectroscopy and multispectral optoacoustic tomography. The complexes retained their biophysical properties, i.e. their ability to seed Aβ fibril formation. They remained stable in biological media for at least 2 days and showed no toxicity to SH-SY5Y neuroblastoma cells up to 1.5 nM and 6 μM of gold nanorods and Aβ seeds, respectively. Taken together, this study describes the first steps in the development of probes for monitoring the spread of Aβ seeds in animal models.

## Introduction

Aggregation and deposition of Aβ and tau proteins occurs in the brain in Alzheimer’s disease and is linked to neurodegeneration [1]. However, the trigger for Aβ and tau deposition remains unknown. Recent research has highlighted pathological similarities between amyloid and prion diseases [2–4]. In prion disease, the mis-folded protein acts as a template or ‘seed’ to convert the native protein to a mis-folded pathogenic form. Growing evidence suggests that a similar ‘seeding’ mechanism could be associated with additional amyloid disorders such as Alzheimer’s disease [3], Parkinson’s disease [5], Huntington’s disease [6], AA amyloidosis [7] and ATTR amyloidosis [8].

The body of evidence supporting Aβ having prion-like properties and Aβ-associated diseases spreading via seeding is rapidly expanding. Early work showed that injection of Alzheimer’s disease brain homogenates into young APP23 mice overexpressing amyloid precursor protein (APP) resulted in accelerated disease pathology observed by the presence of Aβ plaques at an earlier age than control animals [4, 9]. Whilst in the initial work, seeds were injected directly into the brain, a further study has shown that peripheral injection of seeds into the peritoneal cavity can also accelerate disease onset [10]. Support for a similar seeding mechanism in humans was reported from post-mortem analysis of a cohort of patients that had received growth hormone injections, derived from cadavers, as children. A sub-set of this cohort developed Creuzt-Feldt Jacob disease (CJD), a well-known prion disease, as a result of growth hormone contamination with prion seeds. In 2015, Jaunmuktane, et al., reported that 4/8 patients also had signs of Aβ deposition consistent with early onset of Alzheimer’s disease and suggested that these patients could have contracted Alzheimer’s disease in a similar mechanism to CJD, i.e. a prion-like infection with Alzheimer’s disease seeds from cadaveric tissue [11], although those results are debated [12–14].

Evidence for the transmission of amyloid proteins is not confined to neurodegenerative diseases. Several groups have demonstrated the transmissibility of AA amyloidosis through a variety of administration routes and across species [15–17]. Furthermore there are suggestions that seeding mechanisms play a role in the development and progression of ATTR amyloidosis which affect treatment and prognosis [8]. In all these diseases and examples, a critical currently unknown factor is how seed species trigger disease, specifically how seed species injected at one site (e.g. peritoneal cavity of mice) can affect amyloid deposition elsewhere in the body (e.g. brain). To investigate this puzzling phenomenon, novel experimental approaches to track seed species from site of injection to site of action are needed. This would result in enhanced understanding of the mechanisms that underpin disease initiation and progression. As a first step towards this ambitious goal, we present in this article the preparation of seeds labelled with gold nanorods for future tracking with multispectral optoacoustic tomography (MSOT).

MSOT is a real-time optical imaging technique that provides a non-ionising and noninvasive imaging modality which relies on the photoacoustic effect, which was first observed by Alexander G Bell in 1880 [18]. MSOT has been used for several preclinical and clinical applications [19–21] due to its high spatial resolution (~150 μm) and penetration depth (~ 5 cm) [22]. In optoacoustic tomography, a short near infrared laser pulse is partially absorbed by endogenous molecules or exogenous contrast agents causing a rapid thermoelastic expansion, which generates ultrasound waves. These are detected by ultrasonic transducers placed outside the tissue [23]. MSOT relies on a multiwavelength excitation and subsequent spectral deconvolution to identify endogenous molecules or exogenous contrast agents [22]. Therefore, agents with strong and distinctive absorption spectra in the near infrared are best suited for MSOT imaging.

Gold nanoparticles have strong optical absorption that results from the surface plasmon resonance. The position of the absorption band can be tuned through changes in nanoparticle size and shape. Gold nanorods exhibit two bands, the transverse plasmon band located in the visible region around 520 nm, and the longitudinal band located in the near infra-red region, with the exact wavelength tunable by controlling the aspect ratio of the nanorods [24]. For this reason, gold nanorods have been successfully used as contrast agents for MSOT [25, 26]. In this work, we cross-link anti-Aβ antibodies to gold nanorods (Abs-GNR) and demonstrate that those conjugates selectively associate with Aβ seeds. The resulting seeds-Abs-GNR complexes can serve as optical probes for MSOT imaging to monitor the propagation of Aβ seeds in *in vivo* murine models.

## Materials and methods

### Materials

Prior to use, all glassware was washed with aqua regia and rinsed thoroughly with milli-Q water. All chemicals were used as received. Hydrogen tetrachloroaurate (III) hydrate (HAuCl_4_), hexadecyltrimethylammonium bromide (CTAB), sodium borohydride (NaBH_4_), ascorbic acid, Ethyl-3-(3-dimethylaminopropyl)carbodiimide (EDC)/N-hydroxysulfo-succinimide (sulfo-NHS), 2-(N-morpholino)ethanesulfonic acid (MES), poly(isobutylene-maleic-altanhydride), hydrochloridric acid, 5-bromosalicylic acid, silver nitrate (AgNO_3_), phosphine buffered saline (PBS), Ethylenediaminetetraacetic acid (EDTA), resazurin, and dodecylamine, α-metoxy-ω-thiol- poly(ethylene glycol) (mPEG-SH (PEG_2k_), Mw = 2 kDa) HAMS-F12, Non-essential amino acids (NEAA), Penicillin-Streptomycin (Pen-Strep), mouse serum and Foetal Bovine Serum (FBS) were purchased from Sigma-Aldrich (UK). All cell culture plasticware was purchased from Corning Inc. (Corning, USA).

### Synthesis of gold nanorods

Gold nanorods with core diameter and length of ~15 and 60 nm, respectively, were prepared by the seed-mediated growth method involving two steps (seed and growth solutions) as reported previously [27]. Briefly, a seed solution (gold nanoparticles smaller than 4 nm diameter) was prepared by mixing 5 mL of 0.5 mM HAuCl_4_ with 5 mL of 0.2 M CTAB in a glass vial. To this solution, 0.6 mL of freshly prepared NaBH_4_ diluted to 1 mL with deionized water was added with strong stirring for 2 min. The resultant brown-yellow color solution was kept at room temperature for 15 min before use. To prepare a growth solution, 9.0 g of CTAB and 1.1 g of 5-bromosalicylic acid were dissolved in 250 mL of warm water in a 500 mL Erlenmeyer flask. After that, the solution was cooled down to 30°C and 18 mL of 4 mM AgNO_3_was added. The mixture was kept at 30°C for 15 min, and then 250 mL of 1 mM HAuCl_4_ solution was added. After 15 min of stirring (400 rpm), 2 mL of 0.064 M of ascorbic acid was added, and the solution was vigorously stirred for 30 s until it became colourless. Finally, 0.4 mL of freshly prepared seed solution (as described above) was injected into the growth solution and stirred for 30 s. Finally, the solution was kept overnight at 30°C without stirring to allow the gold nanorods to grow. Next day, the resultant gold nanorods were subjected to one-time cleaning by centrifugation (8960 g, 30 min). Thereafter, the supernatant was discarded and the pellet was dispersed in deionised water and stored for further use.

### Phase transfer and polymer coating

For polymer coating of gold nanorods, the aqueous solution was first subjected to a phase transfer from aqueous medium to organic solvent following the protocol previously reported [28]. Briefly, a specific amount of PEG_2k_ in water (S1 Table) was injected into the gold nanorod solution and stirred at room temperature for 24 h. After that, a 0.4 M solution of dodecylamine in chloroform was added and the mixture stirred at 1200 rpm prior to transfer of gold nanorods to the chloroform phase. For purification, the particles were centrifuged twice (see S1 Table for parameters) to remove unbound/unreacted dodecylamine and PEG_2k_. The gold nanorods were dispersed in chloroform and stored for further use. Their concentration was calculated using Beer Lambert law (A_450_ = c_NP_ · ε(450 nm) · l_path_) with UV/Vis absorbance determined at wavelength 450 nm (A_450_) and extinction coefficient (ε(450 nm) = 2.11·10^9^ [M^-1^ cm^-1^]) as reported for gold nanorods of very similar dimensions [29]. Measurements were carried out with a cuvette path length of l_path_ = 1 cm.

**Scheme 1:**
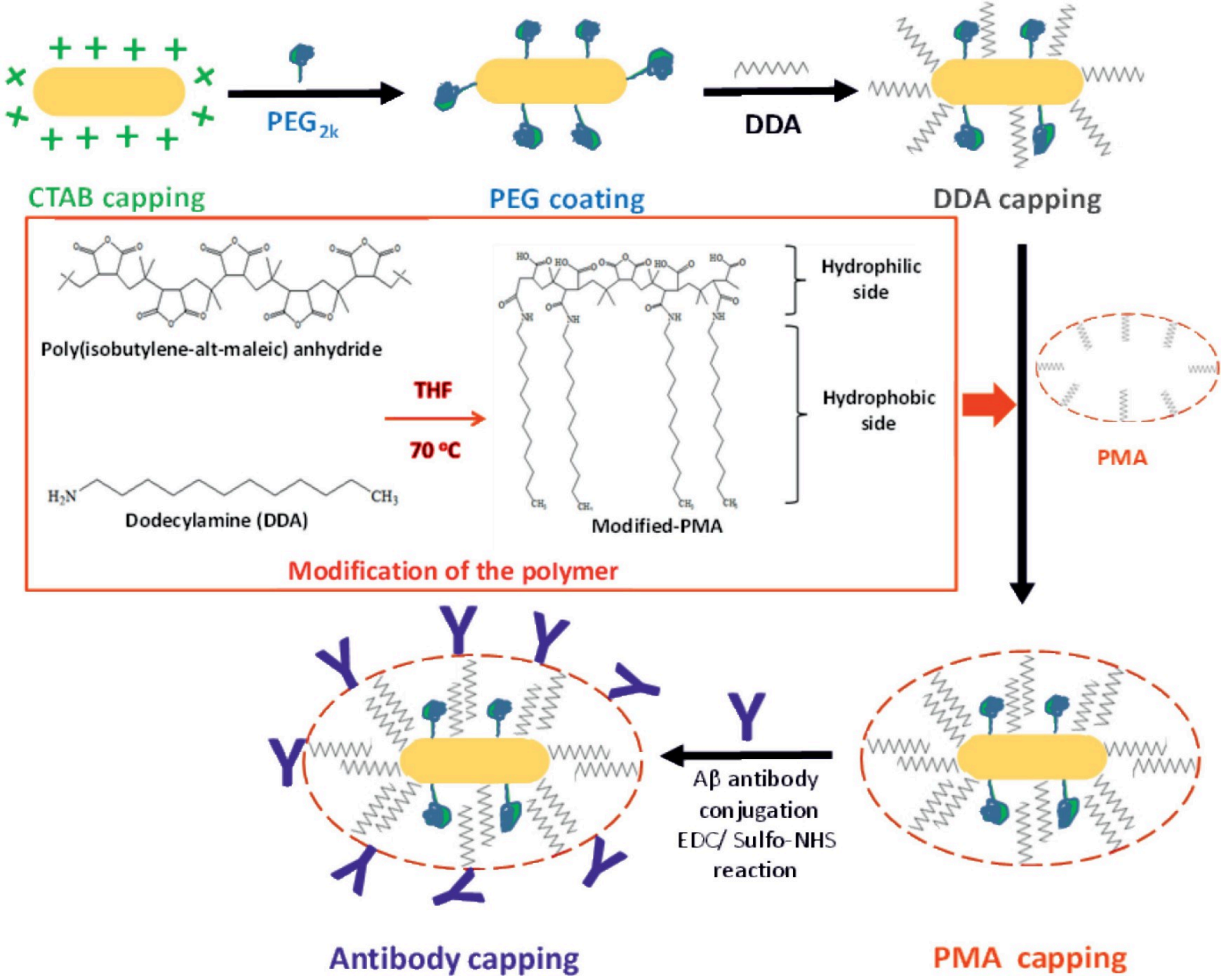
Surface modification.

The dodecylamine-capped gold nanorods were transferred from chloroform to water by applying a polymer coating technique using dodecyl–grafted-poly(isobutylene-alt-maleic anhydride)(in the following referred to as PMA) as described in [30]. For details about the synthesis of the amphiphilic polymer used here we refer to previous work [30]. The amount of added PMA per nanoparticle (NP) was calculated as described in S1 Appendix. The polymer coating was then carried out according to the protocol previously described [28]. Scheme 1 shows the steps of NPs’ surface modification.

### Gold nanorods functionalisation with antibodies

Following polymer coating with PMA, the gold nanorods’ surface is rich in carboxylic groups (–COOH) which can be linked to Aβ antibodies (Abs-Aβ) using EDC/sulfo-NHS coupling reaction (Scheme 1) [31]. To achieve high conjugation efficiency, the concentrations of coupling reagents (EDC/Sulfo-NHS) and Abs-Aβ were optimised sequentially. The molar ratio between EDC and sulfo-NHS was fixed at 1:2, and the amount of Abs-Aβ added to the reaction was introduced with the same volume as described in the following coupling reaction. Briefly, 10 μL of Abs-Aβ (5–75 μg/mL, 6E10 BioLegend) was added to a suspension of gold nanorods at a concentration of 5 nM in 10 mM MES buffer at pH 6. To this mixture, an equal volume of a solution containing 0.5–4 mmoles of sulfo-NHS and 1–8 mmoles of EDC was added (see S2 Table for more details). The reaction mixture was left at room temperature for 2 h, followed by incubation for 6 h at 4°C.

### Association of gold nanorods with Aβ40

Aβ40 (BioLegend, UK) was incubated in phosphate buffered saline (PBS) at 200 μM with agitation for 3 days at 37°C to form fibrils. To produce seeds, Aβ fibrils were subjected to three cycles of ultrasonication pulses (amplitude of 10%) for a period of 30 s/cycle. Aβ fibrils/seeds were diluted to required concentrations (0.1–10 μM equivalent monomer concentration) and incubated with gold nanorods (5–500 pM) overnight at room temperature before use. The resulted complex (Aβ fibrils or Aβ seeds-GNRs) were characterized by UV/Vis spectroscopy and transmission electron microscope (TEM).

### Physicochemical characterisation

Absorption spectra of gold nanorods with different surface coatings were acquired across the wavelength range of 400–1000 nm using a UV/Vis spectrometer (Cary Eclipse).

A Malvern zetasizer Nano ZS was used to measure the hydrodynamic diameter (d_h_) and zeta potential (ζ-potential) of the gold nanorods in water with dynamic light scattering and laser Doppler anemometry respectively. The latter was also used to characterize the ζ-potentials of the prepared Aβ-seeds. All samples were equilibrated for 5 min at 25°C to ensure motion was due to Brownian motion and not due to any thermal gradients. The data was acquired at 173° backscatter settings, using a 633 nm laser. The determined d_h_ [nm] and ζ-potentials [mV] are summarized in S3 Table. Each reported value was the average of at least three independent measurements.

Gold nanorods were also characterised at each step of surface modification by analytical disc centrifugation using a DC24000 instrument (CPS Instruments Inc.). Analytical disc centrifugation separates the nanoparticles according to their size and density under applied centrifugal force. When the nanoparticles are functionalised with a new conjugate, the particle sedimentation size will be altered (compared to non-conjugated NPs) due to the change in the overall density. Analytical disc centrifugation measures the particle size distribution by directly correlating the change in the NP sedimentation time to the change in its overall density. Therefore, analytical disc centrifugation can track changes in the particle size after each step of surface modification. For this purpose, a gradient fluid, 8–24 wt% sucrose solution in Milli-Q water was freshly prepared and injected in consecutive steps into the disc, rotating at a speed of 22,000 rpm. Calibration was performed using poly(vinyl chloride) particles (PVC, 0.377 μm, Analytik Ltd., UK) as a calibration standard before each measurement. Following PVC measurement, 100 μL of gold nanorods solution was injected and analysed three times to assess reproducibility.

Gold nanorod dimensions, surface modification and interaction with Aβ species were investigated by TEM (Tecnai G3 spirit). Samples were prepared by deposition of a drop (5 μL) of diluted solution on a carbon coated copper grid and left to dry at room temperature. For negative staining, a drop of gold nanorod solution (incubated with Aβ-fibrils/seeds) was placed on a carbon-coated copper grid for 2 min. Following blotting, 5 μL of 4% uranyl acetate was added for 30 s. Size distributions were determined using ImageJ. At least 100 particles were considered to determine the average size of the different cores.

Gel electrophoresis experiments were conducted using 1% agarose gels prepared in tris-EDTA buffer at pH 7.4. Gold nanorod solutions were mixed with negatively charged orange G as a gel-loading buffer to increase the density of the sample prior to loading in the gel wells, following the protocol reported in [28]. Samples were run under an electric field of 100 V in tris-EDTA buffer at room temperature for 1 h using a Bio-Rad horizontal electrophoresis system.

### Gold nanorods effect on protein aggregation

Thioflavin T (ThT) fluorescence assay was used to assess fibril formation using a FlexStation3 Multi-Mode Microplate Reader (Molecular Devices). 10 μM Aβ40 with 2 μM of ThT was incubated in PBS at 37°C in a clear bottom/black wall 96 well plate. Measurements were taken every 5 min with shaking for 5 s before each read with excitation and emission at 440 nm and 480 nm, respectively. To stop the depletion of Aβ40 from solution resulting from non-specific binding, the plates were pre-coated with polyethylene glycol [32]. Briefly, 300 μL of 0.01 M polyethylene glycol (Mw = 300 g/mol) was added into each well and incubated at room temperature for 60 min. The wells were then aspirated and rinsed with 10 times their volume (3 ml) of Millipore ultra-pure water. The plates were allowed to dry at room temperature before use.

### Cell viability

Cell viability was tested using SH-SY5Y neuroblastoma cells at cell density of 10,000 cells/well in a 96 well plate. Solutions of Abs-GNRs and Aβ seeds were mixed overnight at room temperature at final concentrations of 25 nM and 100 μM, respectively. The same concentrations from stocks of PMA-GNRs, Abs-GNRs, or Aβ seeds, were obtained. Cells were incubated with a dilution series of the particles/Aβ proteins for 24 h and viability evaluated using resazurin assay [33]. Resazurin is a blue, non-fluorescent sodium salt, which is converted to Resorufin by metabolically active cells. Fluorescence spectra were recorded using a FlexStation3 Multi-Mode Microplate Reader with excitation wavelength of 560 nm. Viability data are presented as percentage compared to untreated cells. Data was statistically analysed using analysis of variance and Tukey’s post hoc test with p<0.05.

### Optoacoustic imaging

MSOT imaging was performed using the MSOT inVision 256-TF small animal imaging system (iThera Medical, Munich, Germany). Agar phantoms were prepared by mixing 4 g of agar in 100 mL of hot water. This solution was placed in 50 mL tubes and stored at 4°C for further use. To prepare gold nanorod containing phantoms, different concentrations of 100 μL of Aβ-seeds-GNRs were placed in phantoms wells. Five cross sections of the phantom were analyzed using 15 different wavelengths in the range from 660 to 950 nm. The phantom was scanned in 2 mm steps using acquisition wavelengths; 680, 720, 760, 800, 810, 820, 830, 840, 850, 860, 870, 880, 890, 900, 950 nm. A linear based reconstruction method was applied using ViewMSOT software (iThera Medical, Munich, Germany). For multispectral images, the signal for the gold nanorods was unmixed by multispectral processing using the absorbance spectra for the gold nanorods.

## Results and discussion

### Gold nanorods synthesis

**Fig 1:**
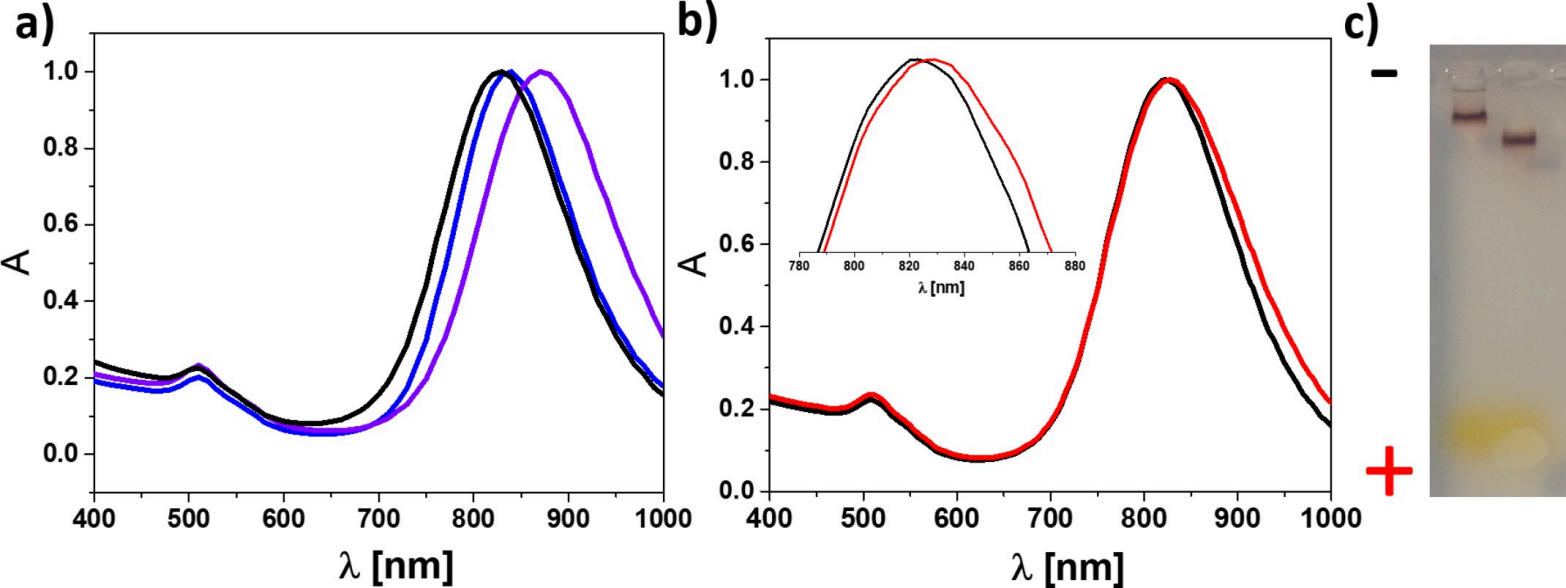
Conjugation of Aβ antibody to gold nanorods. a) Normalised absorption spectra for CTAB-capped gold nanorods (in water, blue), dodecylamine-capped gold nanorods (in chloroform, violet) and PMA-coated gold nanorods (in water, black). b) Normalised absorption spectra for gold nanorods after PMA coating (PMA-GNRs, black) and after conjugation with Aβ antibody (Abs-GNRs, red). Inset shows normalized data for the LSPR shift of Abs-GNRs. c) Gel electrophoresis of gold nanorods before (right lane, PMA-GNRs) and after (left lane, Abs-GNRs) conjugation with Aβ antibody.

Gold nanorods were prepared using a well-established procedure that results in particles capped with the toxic cationic surfactant CTAB [27]. To remove the bound CTAB, the gold nanorods were transferred from water to chloroform using both PEG_2k_ and dodecylamine. Gold nanorods exhibit characteristic absorbance peaks in both the visible and near IR regions. The positions of those peaks are sensitive to the size and geometry of the particle, but also to its local environment refractive index. Thus, a 40 nm red shift of the longitudinal plasmon band was observed (from 830 nm to 870 nm) due to the change in the refractive index of the dispersion medium from water to chloroform as shown in Fig 1A. Coating with an amphiphilic polymer (PMA-GNRs) was then used to transfer the particles back from chloroform to water and to serve as a platform for immobilisation of antibodies using the water-soluble cross-linkers EDC and sulfo-NHS [30]. The spectrum after transfer is similar to the starting material with a small blue shift of the peak. A range of experimental conditions were tested to maximise coupling yield estimated by the red shift of the plasmon band (S2 Table and S1 Fig); in the optimised conditions a ∼5 nm red shift is observed (Fig 1B, inset) [34]. In the absence of EDC/sulfo-NHS no change in localised surface plasmon resonance (LSPR) was detected consistent with no binding occurring (S1A Fig). Electron microscopy and dynamic light scattering confirmed that the particles have a narrow size distribution that is preserved during the various modification steps, with a final increase of hydrodynamic diameter upon coupling of the Aβ antibody (S2 and S3 Figs and S3 Table). Consistent with previous work [35, 36], the conjugation results in a reduction in the apparent diameter measured by analytical disc centrifugation due to reduction in overall density upon antibody binding (S4 Fig). Coupling of the Aβ antibody to the PMA-GNRs was further confirmed by reduced electrophoretic mobility (Fig 1C) and changes in zeta potential (S5 Fig and S3 Table).

### Abs-GNRs interact specifically with Aβ40

**Fig 2:**
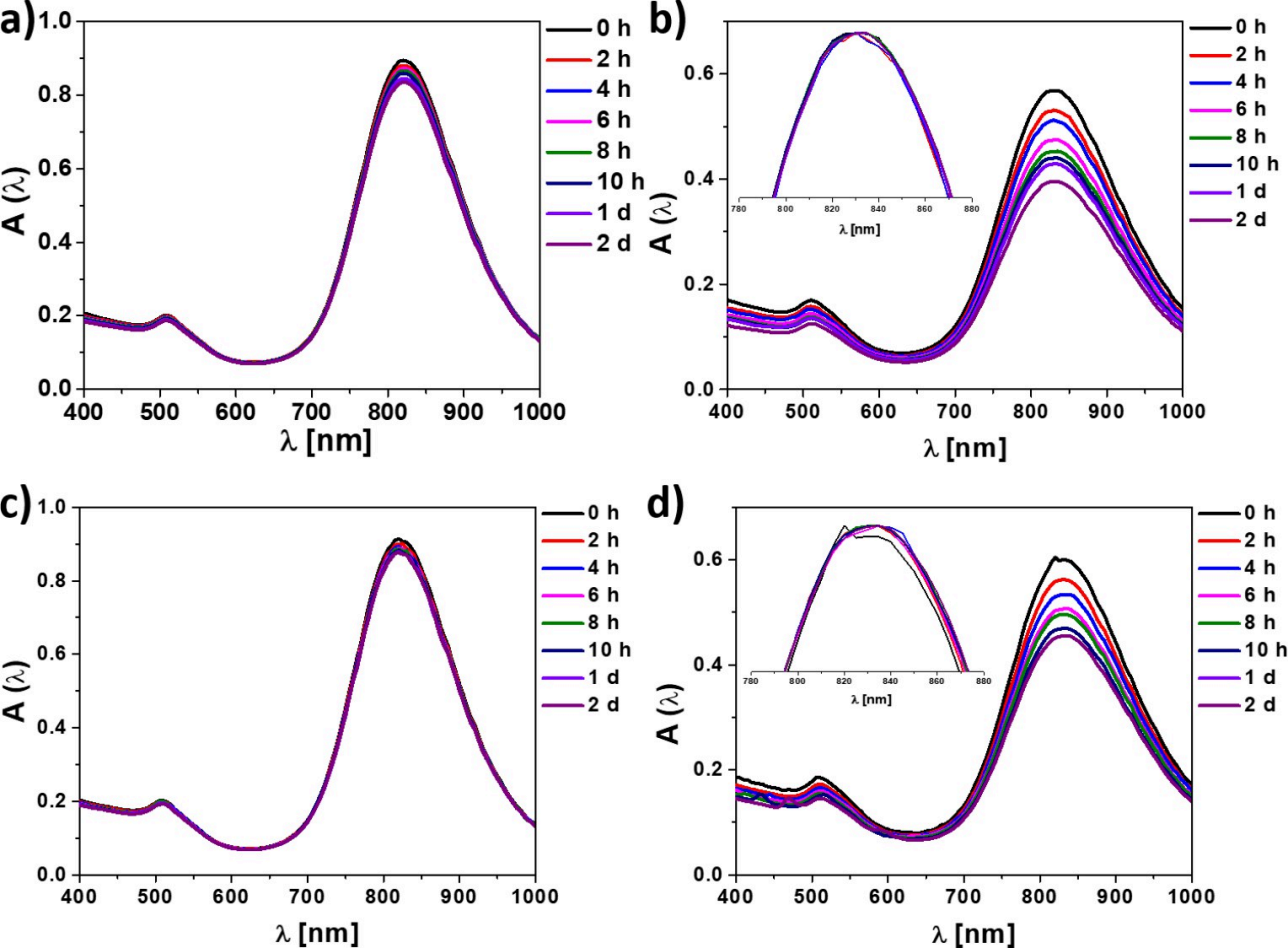
Abs-GNRs show specific association with Aβ40 fibrils and seeds. Absorption spectra (not normalised) of a) PMA-GNRs with Aβ-seeds, b) Abs-GNRs with Aβ-seeds, c) PMA-GNRs with Aβ fibrils, and d) Abs-GNRs with Aβ fibrils in water at different time-points as shown. Loss of intensity is observed due to conjugation with insoluble Aβ seeds and fibrils. Insets show normalised spectra.

**Fig 3:**
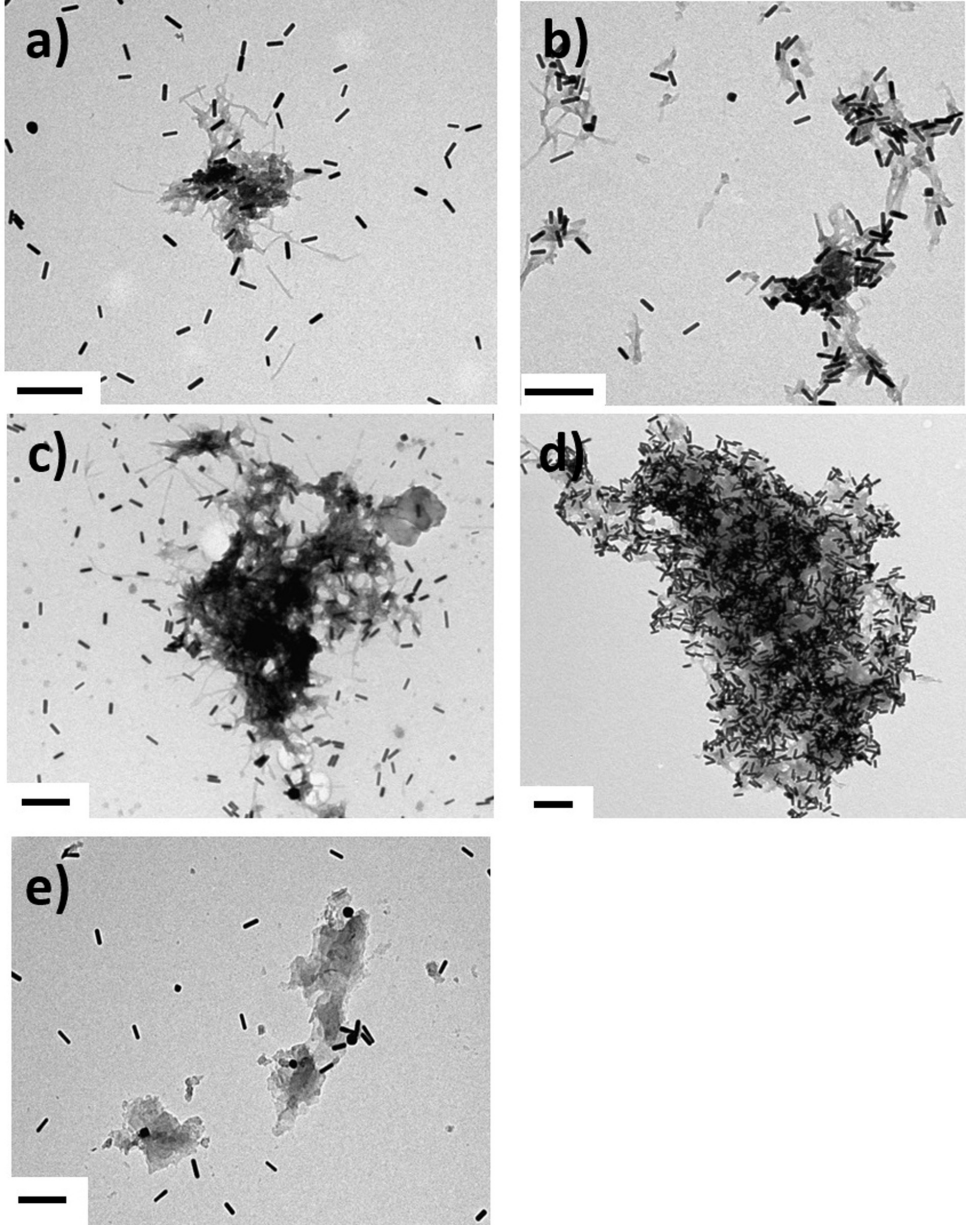
Electron microscopy confirms specific association of Abs-GNRs with Aβ seeds and fibrils. Aβ seeds incubated overnight with a) PMA-GNRs and b) Abs-GNRs. Aβ fibrils incubated overnight with c) PMA-GNRs and d) Abs-GNRs. e) Abs-GNRS incubated overnight with seeds formed from a control amyloidogenic protein medin. Scale bars correspond to 200 nm.

The interaction of PMA-GNRs and Abs-GNRs with Aβ was investigated by analysing changes in the absorbance spectra of the gold nanorods in the presence of Aβ seeds and fibrils. No changes in the longitudinal plasmon band were observed when PMA-GNRs were incubated with Aβ seeds or fibrils (Fig 2A and 2C). In contrast, with Abs-GNRs the spectra indicated a progressive reduction in intensity indicative of association with seeds and fibrils (Fig 2B and 2D). As the Fig insets show, the position of the plasmon band maxima do not shift significantly. This absence of plasmon coupling indicates that the gold nanorods remain separated from each other within the fibrillar networks [37]. This may be explained by two main factors: 1) the large steric barrier between the nanorod cores provided by the PMA and antibody layers; 2) the nanorods do not bind directly to each other but instead to another extended object, i.e. the seeds or fibrils. The preserved shape of the spectrum is an important advantage for future MSOT detection as this technique relies on multispectral unmixing to distinguish contrast agents from endogenous absorbers. Specific association of Abs-GNRs with Aβ40 was confirmed by TEM where Abs-GNRs were almost entirely co-localised with Aβ seeds and fibrils, whereas under the same conditions, a large proportion of PMA-GNRs were observed randomly scattered over the grid (Fig 3). In the representative images shown in Fig 3, ~95% of Abs-GNRs colocalize with seeds compared with only ~18% of PMA-GNRs. Additional images are shown in S6 Fig. Incubation of Abs-GNRS overnight with seeds from a different amyloidogenic protein medin confirmed that the association between Abs-GNRs and Aβ is specific (Fig 3E).

### Seeds-Abs-GNR complexes are stable and non-toxic

**Fig 4:**
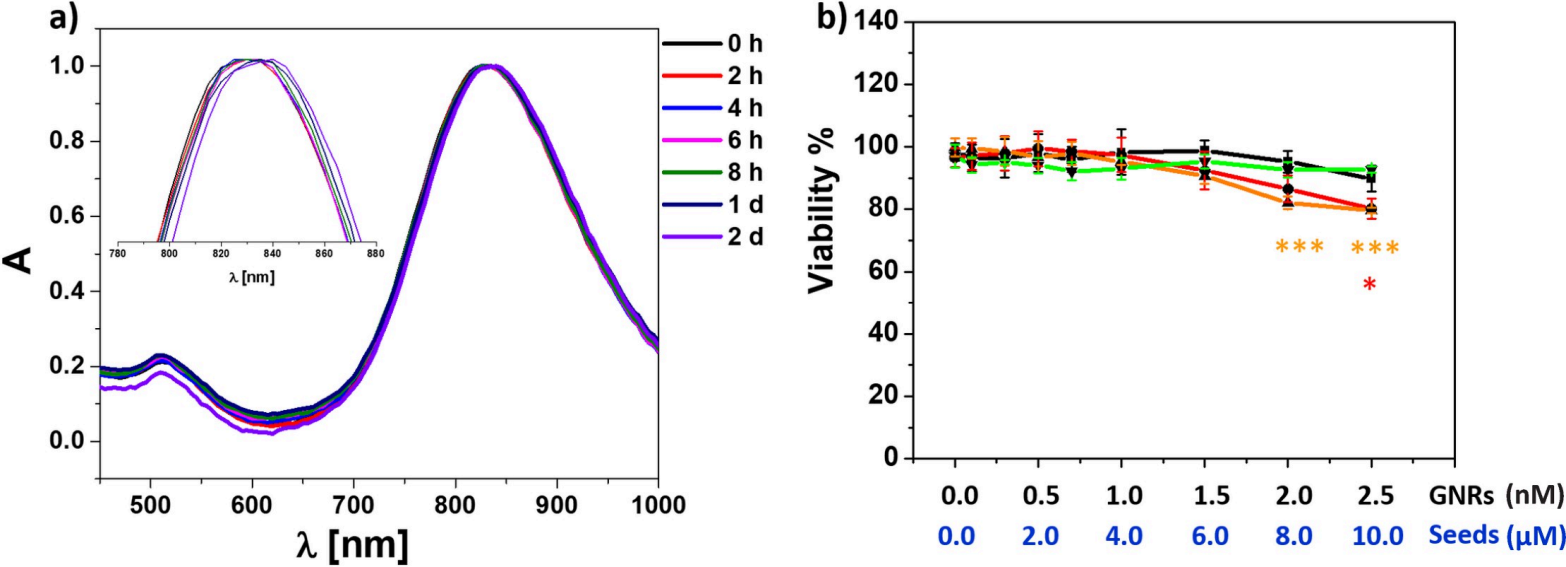
Gold nanorod complexes show stability and no reduction in cell viability for seeds-Abs-GNRs containing up to 1.5 nM GNRs and 6 μM seeds. (a) Normalised absorption spectra for pre-formed seeds-Aβ-GNRs in mouse serum at different time points (up to 2 d). (b) Cellular viability (%) as determined by the resazurin assay with SHSY5Y cells exposed to PMA-GNRs (black), Abs-GNRs (red), seeds-Abs-GNRs (orange) and Aβ-seeds alone (green) for 24 h at the indicated concentrations of gold nanorods (nM) and Aβ seeds (μM). Data presented as mean ± SD from 3 wells per condition and analysed statistically using analysis of variance and Tukey’s post hoc test with p<0.05. *p<0.05, ***p<0.0001 compared to cells alone with colour corresponding to condition tested. Raw data is available in S1 Data.

Seeds-Abs-GNR complexes showed stability assessed by absorption spectra in mouse serum, water and cell media up to 2 days (Figs 4A and S7). To test viability, SH-SY5Y cells were exposed to PMA-GNRs, Abs-GNRs, seeds-Abs-GNRs and Aβ seeds for 24 h with viability assessed using the resazurin assay (Fig 4B). Results showed that the presence of seeds-Abs-GNRs containing 1.5 nM GNRs and 6 μM seeds (Fig 4B, orange) can be employed in the developed optical probe with no significant effect on cell viability under conditions used. These results are in good agreement with previous studies on toxicity of gold nanorods [28, 38] and Aβ [39].

### Seeds-Abs-GNR-complexes retain seeding ability and can be integrated into fibrillar networks

**Fig 5:**
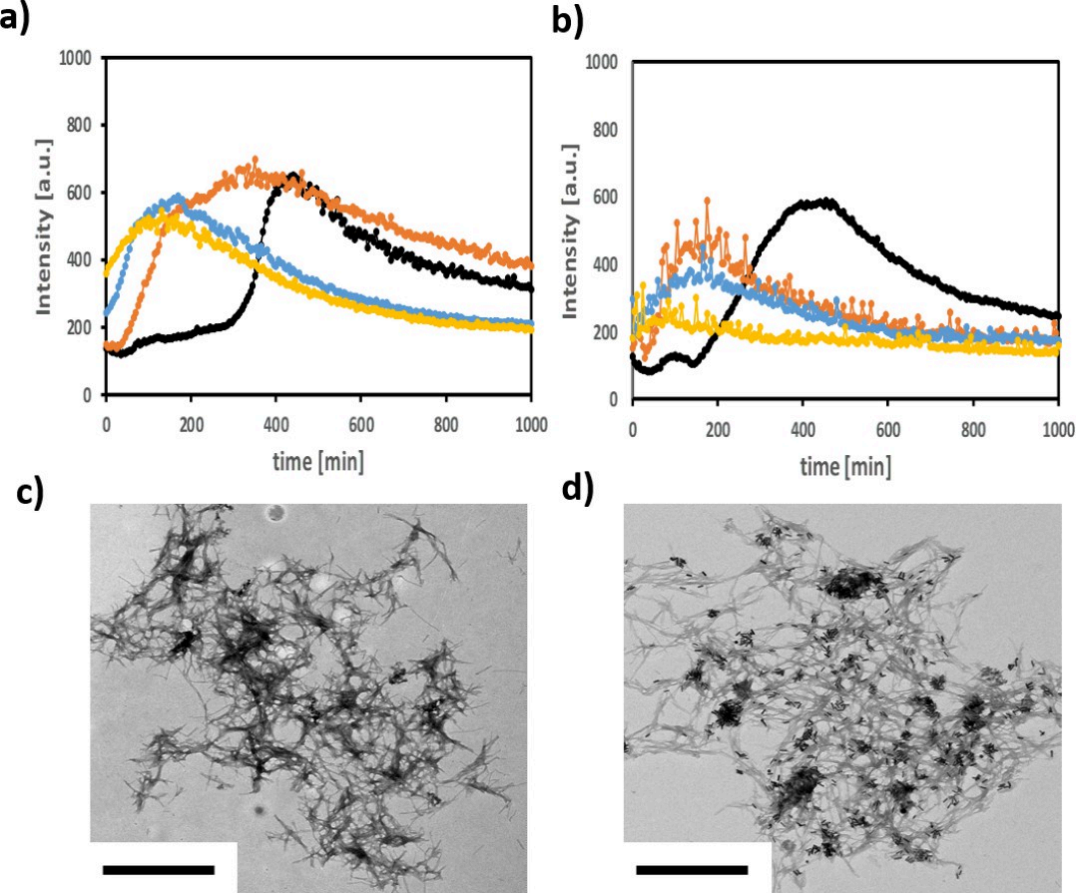
Seeds-Abs-GNRs complexes retain seeding ability and can be integrated into fibrillar networks. a) Aggregation kinetics assessed by ThT fluorescence of 10 μM Aβ40 in PBS (pH 7.4, 37°C) in absence (black) or presence of seeds at varying concentrations; 0.1 μM (orange), 0.5 μM (light blue), and 1 μM (yellow). b) ThT fluorescence of 10 μM Aβ40 incubated in absence (black) or presence of pre-formed seeds-Abs-GNRs formed from 0.4 nM of Abs-GNRs with increasing concentrations of seeds as above 0.1 μM (orange), 0.5 μM (light blue), and 1 μM (yellow). TEM images of 10 μM Aβ40 incubated in PBS, pH 7.4 for 10 h at 37°C in the presence of c) plain seeds and d) seeds-Abs-GNRs, confirming that seeds-Abs-GNR complexes are integrated within fibrillar networks. Scale bars correspond to 1 μm.

The formation of Aβ40 fibrils is nucleation-dependent and addition of pre-formed Aβ40 seeds resulted in a concentration-dependent reduction in lag time (Fig 5A). Addition of seeds-Abs-GNR complexes showed a similar concentration-dependent reduction in lag time confirming they have retained the ability to seed Aβ40 fibrillation (Fig 5B). Addition of PMA-GNRs had no effect on fibril formation (S8 Fig) consistent with lack of association between PMA-GNRs and Aβ40. Addition of Abs-GNRs in the absence of pre-formed seeds resulted in increased Aβ40 fibrillation lag time and reduced fibril fluorescence intensity (S8 Fig) which may be caused by depletion of Aβ from solution via binding to Abs-GNRs. TEM was used to confirm that Aβ fibrils formed in the presence or absence of seeds-Abs-GNRs show no morphological differences with gold nanorods observed within fibrillar networks, suggesting incorporation of bound seeds during the fibrillation process (Fig 5C and 5D).

### Optoacoustic imaging of seeds-Abs-GNR complexes

**Fig 6:**
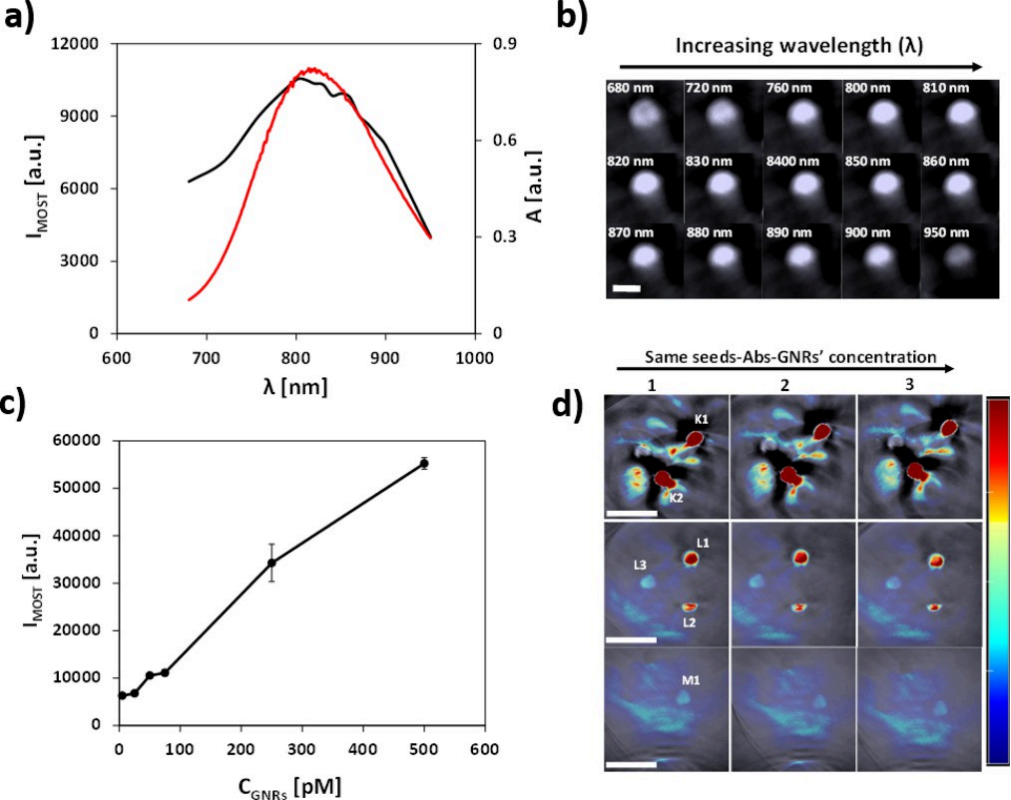
Phantom MSOT experiments. a) Comparison of MSOT intensity signals (black) and UV spectrum (red) of seeds-Abs-GNRs agar phantom. b) Corresponding single wavelength MSOT images (680–950 nm). Scale bar is 3 mm for all images. c) Mean MSOT signal intensities collected from 3 different positions for increasing concentrations of seeds-Abs-GNRs (5–500 pM). Raw data is available in S1 Data. d) Corresponding multispectral unmixed MSOT images. Concentration of seeds-Abs-GNRs; Top-500 pM (K1), 250 pM (K2), Middle-75 pM (L1), 50 pM (L2), 25 pM (L3), Bottom-5 pM (M1). Images were reconstructed from raw signal data captured with the MSOT system at 3 different cross-sections at 3 different positions in the same phantom (1, 2 & 3) with a logarithmic colour bar. Scale bars are 10 mm. Colour scale range is 1.2 × 10^3^ to 1.2 × 10^4^ MSOT au (same scale is used in all MSOT images).

MSOT data was collected using agar phantoms to test sensitivity and optical properties of seeds-Abs-GNR complexes. A range of seeds-Abs-GNR complexes concentrations (5–500 pM) were imaged and recorded at wavelengths 680 to 950 nm. As expected, the strength of the photoacoustic signal detected by MSOT correlated with the absorbance over this range of wavelengths (Fig 6A) and was associated with a gradual change in the MSOT signal intensity at different wavelengths as shown in the single wavelength images (Fig 6B). The MSOT signal was found to be linearly dependent on concentration (Fig 6C). At 5 pM, the lowest tested concentration, the unmixed MSOT signal is still detectable separately from the background noise of the agar phantom (Fig 6D) which is encouraging for future applications. Previous studies injected Aβ seeds at concentrations of 10–20 ng/μl [10]. We have made our Aβ-Abs-GNR complexes consistent with this concentration of ~2 μM Aβ and 500 pM gold nanorods. MSOT data suggests that even with dilution of seeds upon injection that may occur, sufficient signal would remain to enable tracking of seeds *in vivo*. Estimating precisely the *in vivo* limit of detection is a highly complex issue because sensitivity is affected by tissue depth, the absorption profiles of various organs, attenuation by tissues, as well as the number of wavelengths selected for acquisition [40, 41]. Further calibration work *in vivo* will be required to precisely establish the amount of Abs-GNRs that can be tracked.

## Conclusions

In this work we have exploited the selectivity of antibodies conjugated to gold nanorods to bind to pre-formed Aβ seeds to design an optoacoustic imaging tool for monitoring the propagation of Aβ seeds *in vivo*. The resulting probes have been characterised to be stable, non-toxic and biocompatible while retaining their ability to seed fibril formation comparable with that observed by seeds alone. Preliminary MSOT data demonstrate that the optical signature of gold nanorods was preserved in the complexes and that signal could be detected at concentrations as low as 5 pM. These complexes provide a suitable optical probe to monitor the propagation of Aβ-seeds by MSOT in an *in vivo* model to contribute to a better understanding of the role of Aβ seeds in Alzheimer’s disease progression.

## Supporting information

S1 Table Experimental conditions used for the phase transfer, polymer coating and anti-Aß anybody conjugation. cNP, cDDA and cPEG refers to the concentration of gold nanorods, dodecylamine and PEG, respectively. Rp/Area refers to the number of PMA monomer added per nm^2^ of effective NP surface. a refers to the centrifugation acceleration (g = 9.81 m/s2) and t refers to the centrifugation time.
(DOCX)

S2 Table EDC/sulfo-NHS coupling reaction of Abs-Aβ to PMA-GNRs under different concentrations of EDC/sulfo-NHS at a fixed concentration of Abs-Aβ (sampled coded "a") or different concentrations of Abs-Aβ at a fixed concentration of coupling reagents (samples coded "b"). The ratio between EDC and sulfo-NHS was always kept constant at 1:2, respectively.
(DOCX)

S3 Table Hydrodynamic diameter dh [nm] in number (dh(N)) and intensity (dh(I)), Z-average (dh(Z)), polydispersity index (PDI) and ζ -potential values (ζ) for gold nanorods along their surface modification and upon conjugation with Aβ seeds. Size values for gold nanorods, although without any physical meaning because they are not spherical NPs, are included to show that the colloidal stability was not compromised in the different steps of the polymer coating.
(DOCX)

S1 Fig Normalized absorption spectra for PMA-GNRs before and after conjugation with Abs-Aβ under different experimental conditions using EDC/sulfo-NHS. a) The concentration of EDC/sulfo- NHS was varied while the concentration of Abs-Aβ was fixed. b) The concentration of Abs-Aβ was varied while the concentration of EDC/sulfo-NHS was kept constant. The experimental conditions are listed in S2 Table.
(TIF)

S2 Fig Intensity distribution of hydrodynamic diameters (I(dh)) for gold nanorods along their surface modification. a) CTAB-capped; b) DDA-capped; c) PMA-coated (PMA-GNRs) and d) after conjugation with anti-Aβ antibody (Abs-GNRs).
(TIF)

S3 Fig TEM images of gold nanorods and their corresponding size distribution histograms after different steps of surface modifications. dc refers to the particle core diameter (left histograms) and Lc refers to particle core length (right histograms). A) CTAB-capped GNRs with dc = 15.31 ± 2.39 nm, and Lc = 60.13 ± 5.33 nm. B) PMA-GNRs with dc = 15.25 ± 1.68 nm, and Lc = 59.90 ± 5.85 nm. C) Abs-GNRs with dc = 14.91 ± 1.97 nm, and Lc = 60.98 ± 7.06 nm. Scale bars correspond to 200 nm.
(TIF)

S4 Fig Apparent size distributions measured by analytical disc centrifugation. CTAB-capped (purple), PMA-GNRs (black) and Abs-GNRs (red). The average values are 29.8 ± 0.2 nm, 22.79 ± 0.12 nm and 20.81 ± 0.18 nm respectively.
(TIF)

S5 Fig ζ-potential distribution (N(ζ)) results during gold nanorods surface modification process. a) CTAB-capped; b) PMA-GNRs; c) Abs-GNRs, d) seeds-Abs-GNRs and e) Aβ seeds alone.
(TIF)

S6 Fig Additional TEM images. a) PMA-GNRs and b) Abs-GNRs after incubation with Aβ seeds overnight. Scale bars correspond to 200 nm.
(TIF)

S7 Fig Normalised absorption spectra. a) PMA- GNRs, b) Abs- GNRs, and c) seeds-Abs-GNRs in water (left) and cell media (right) at different point of times (up to 2 days).
(TIF)

S8 Fig PMA-GNRs do not bind to or alter Aβ40 fibrillation. ThT fluorescence of 10 μM Aβ40 in PBS (pH 7.4, 37°C) alone (black), and in the presence of PMA-GNRs (light blue) or Abs-GNRs (orange).
(TIF)

S1 Appendix Calculating the amount of PMA needed for phase transfer of NPs from organic solvent to aqueous medium. (DOCX)

S1 Data Data used to calculate mean ±SD values for Figs 4B and 6C. (XLSX)
