# Supplementary material for: Development of amyloid beta gold nanorod aggregates as optoacoustic probes
Source: PLoS One. 2022 Mar 25;17(3):e0259608. doi: 10.1371/journal.pone.0259608 (PMC8956182; doi:10.1371/journal.pone.0259608)
Supplement: S1 Table — cNP, cDDA and cPEG refers to the concentration of gold nanorods, dodecylamine and PEG, respectively. Rp/Area refers to the number of PMA monomer added per nm2 of effective NP surface. a refers to the centrifugation acceleration (g = 9.81 m/s2) and t refers to the centrifugation time. (DOCX) [file pone.0259608.s001.docx]

| **Sample** | **Phase transfer** | | **Polymer coating** | | |
| --- | --- | --- | --- | --- | --- |
|  | **c_PEG_/ c_NP_** | **c_DDA_[M]** | **R_P_/_Area_ [nm^-2^]** | **Centrifugation** | |
|  |  |  |  | **a [g]** | **t [min]** |
| GNRs | 3 · 10^4^ | 75 | 3000 | 8960 | 30 |
