# Supplementary material for: Development of amyloid beta gold nanorod aggregates as optoacoustic probes
Source: PLoS One. 2022 Mar 25;17(3):e0259608. doi: 10.1371/journal.pone.0259608 (PMC8956182; doi:10.1371/journal.pone.0259608)
Supplement: S2 Table — The ratio between EDC and sulfo-NHS was always kept constant at 1:2, respectively. (DOCX) [file pone.0259608.s002.docx]

| **Condition** | **Abs-Aβ**  **[µg/ mL]** | **EDC**  **[mmoles]** | **sulfo-NHS**  **[mmoles]** | **LSPR shifting**  **[nm]** |
| --- | --- | --- | --- | --- |
| 1a | 0 | 0 | 0 | Control |
| 2a | 50 | 0 | 0 | 0 |
| 3a |  | 0.5 | 1 | ∼2 |
| 4a |  | 1 | 2 | ∼3 |
| **5a** |  | **2** | **4** | ∼**5** |
| 6a |  | 4 | 8 | ∼5 |
| 1b | 0 | 0 | 0 | Control |
| 2b | 5 | 2 | 4 | 0 |
| 3b | 20 |  |  | ∼2 |
| **4b** | **50** |  |  | ∼**5** |
| 5b | 75 |  |  | ∼5 |
