# Supplementary material for: Development of amyloid beta gold nanorod aggregates as optoacoustic probes
Source: PLoS One. 2022 Mar 25;17(3):e0259608. doi: 10.1371/journal.pone.0259608 (PMC8956182; doi:10.1371/journal.pone.0259608)
Supplement: S3 Table — Size values for gold nanorods, although without any physical meaning because they are not spherical NPs, are included to show that the colloidal stability was not compromised in the different steps of the polymer coating. (DOCX) [file pone.0259608.s003.docx]

| **Sample** | **Coating** | **d_h,N_ [nm]** | **d_h,Z_ [nm]** | **PDI** | **d_h,I1_ [nm]** | **d_h,I2_ [nm]** | **ζ [mV]** |
| --- | --- | --- | --- | --- | --- | --- | --- |
| **GNRs** | CTAB | 3.65 ± 0.05 | 7.87 ± 0.25 | 0.3 ± 0.01 | 6.62 ± 0.01 | 99.49 ± 1.65 | +34.1 ± 1.15 |
|  | DDA | 2.75 ± 0.03 | 8.0 ± 0.19 | 0.3± 0.01 | 4.77 ± 0.02 | 79.82 ± 1.06 | - |
|  | PMA | 3.90 ± 0.1 | 10.38 ± 0.1 | 0.4 ± 0.008 | 7.69 ± 0.16 | 89.54 ± 1.87 | -28.8 ± 0.26 |
|  | Abs | 4.5 ± 0.3 | 12.29 ± 0.09 | 0.4 ± 0.006 | 8.25 ± 0.2 | 97.54 ± 1.2 | -17.7 ± 1.75 |
| **Aβ Seeds** | - | - | - | - | - | - | - 44.2 ± 1.10 |
| **Seeds-Abs-GNRs** | - | - | - | - | - | - | - 43.7 ± 0.30 |
