# Supplementary figures and images for: Development of amyloid beta gold nanorod aggregates as optoacoustic probes
Source: PLoS One. 2022 Mar 25;17(3):e0259608. doi: 10.1371/journal.pone.0259608 (PMC8956182; doi:10.1371/journal.pone.0259608)

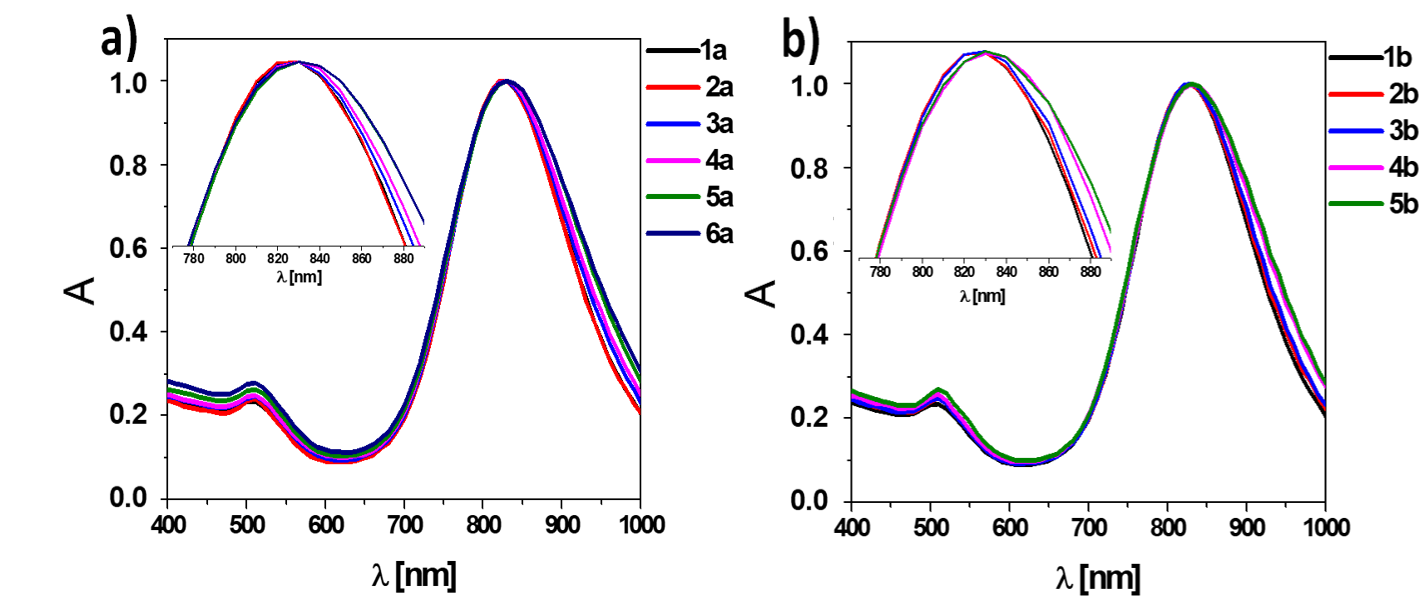

Supplement: S1 Fig — a) The concentration of EDC/sulfo- NHS was varied while the concentration of Abs-Aβ was fixed. b) The concentration of Abs-Aβ was varied while the concentration of EDC/sulfo-NHS was kept constant. The experimental conditions are listed in S2 Table. (TIF) [file pone.0259608.s004.tif]

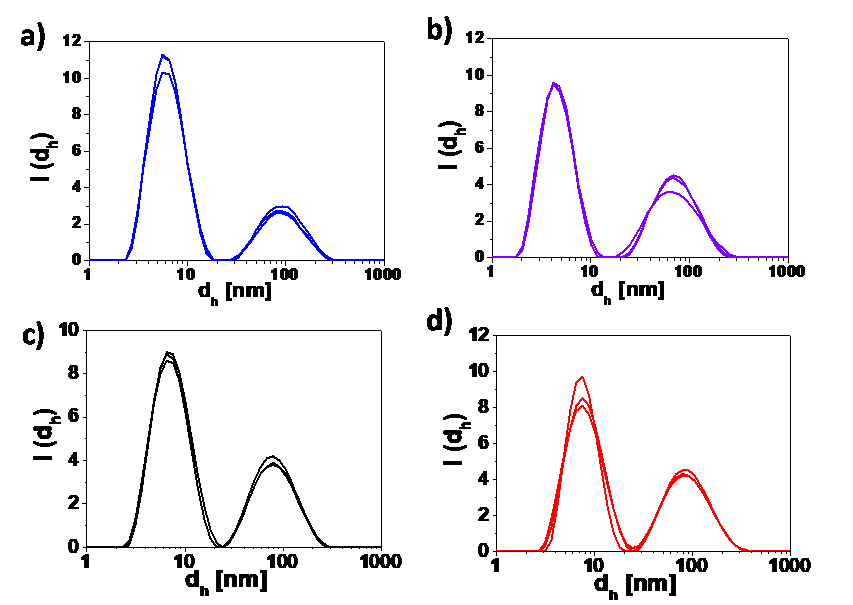

Supplement: S2 Fig — a) CTAB-capped; b) DDA-capped; c) PMA-coated (PMA-GNRs) and d) after conjugation with anti-Aβ antibody (Abs-GNRs). (TIF) [file pone.0259608.s005.tif]

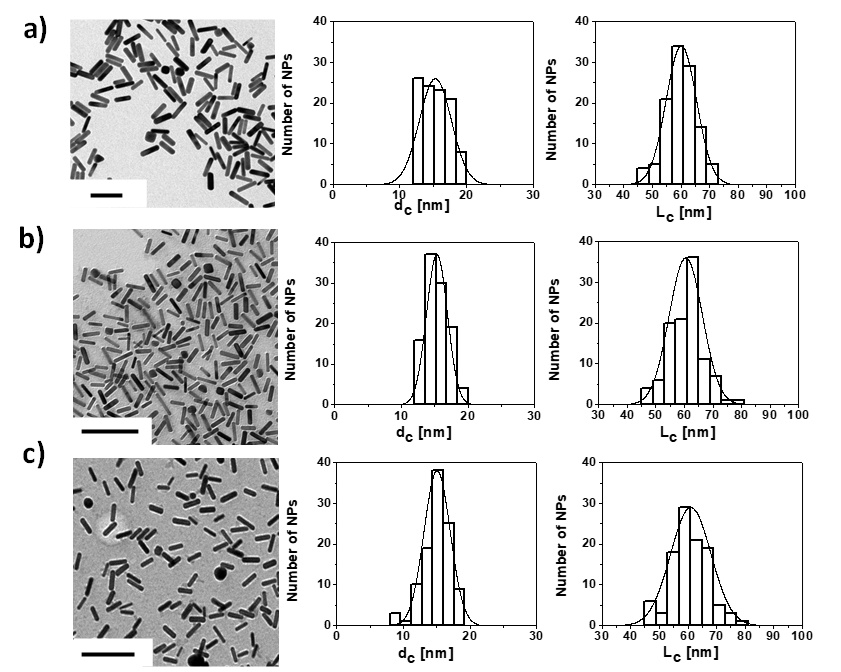

Supplement: S3 Fig — dc refers to the particle core diameter (left histograms) and Lc refers to particle core length (right histograms). A) CTAB-capped GNRs with dc = 15.31 ± 2.39 nm, and Lc = 60.13 ± 5.33 nm. B) PMA-GNRs with dc = 15.25 ± 1.68 nm, and Lc = 59.90 ± 5.85 nm. C) Abs-GNRs with dc = 14.91 ± 1.97 nm, and Lc = 60.98 ± 7.06 nm. Scale bars correspond to 200 nm. (TIF) [file pone.0259608.s006.tif]

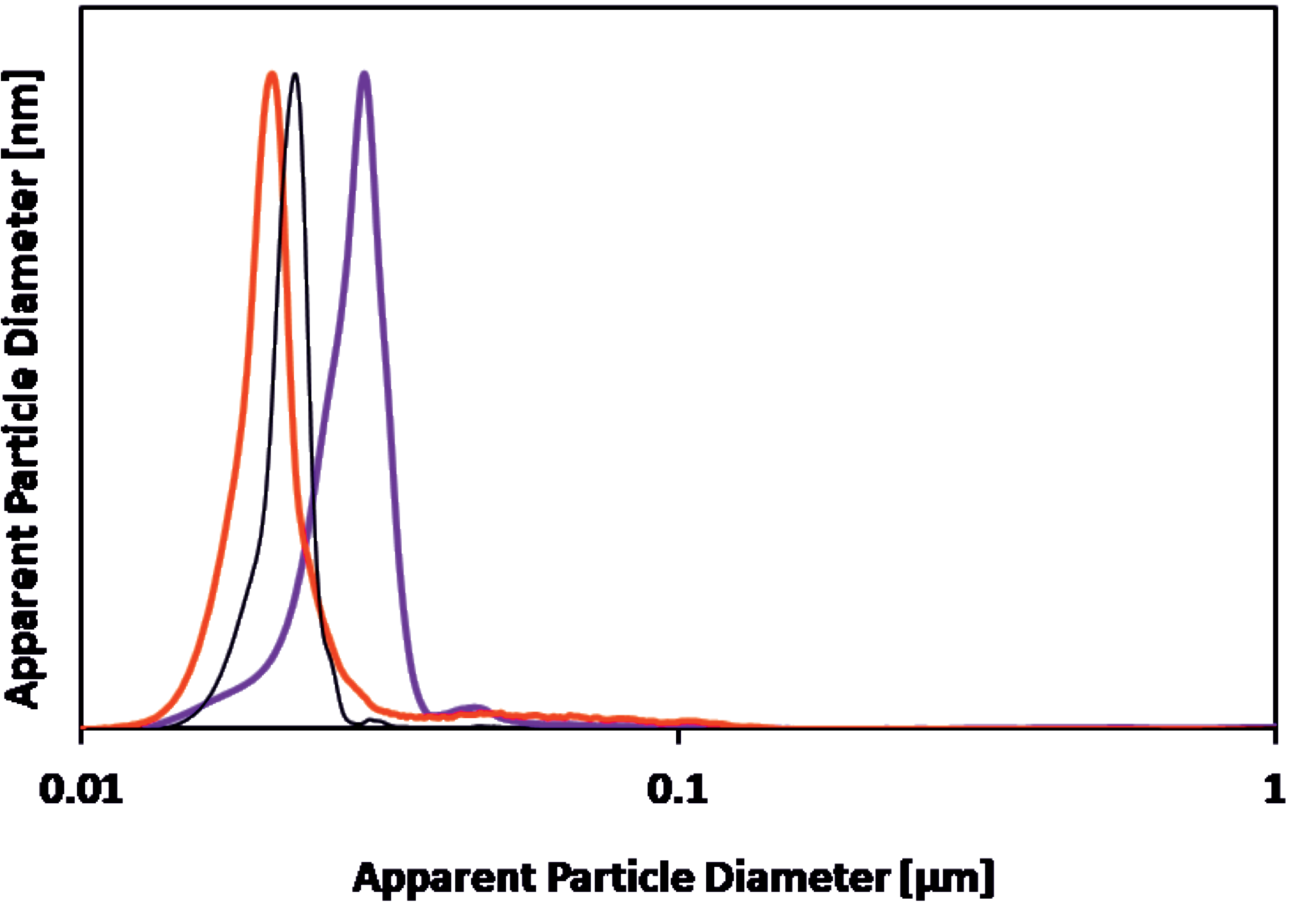

Supplement: S4 Fig — CTAB-capped (purple), PMA-GNRs (black) and Abs-GNRs (red). The average values are 29.8 ± 0.2 nm, 22.79 ± 0.12 nm and 20.81 ± 0.18 nm respectively. (TIF) [file pone.0259608.s007.tif]

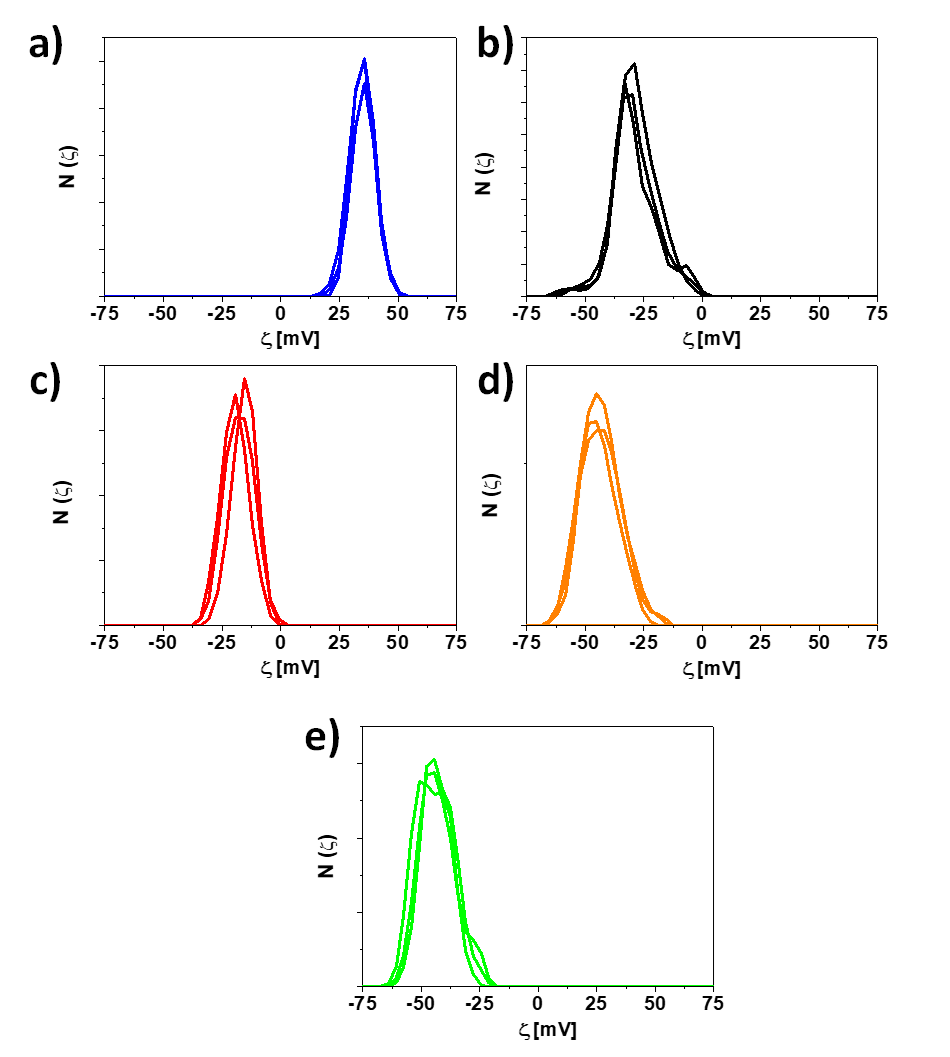

Supplement: S5 Fig — a) CTAB-capped; b) PMA-GNRs; c) Abs-GNRs, d) seeds-Abs-GNRs and e) Aβ seeds alone. (TIF) [file pone.0259608.s008.tif]

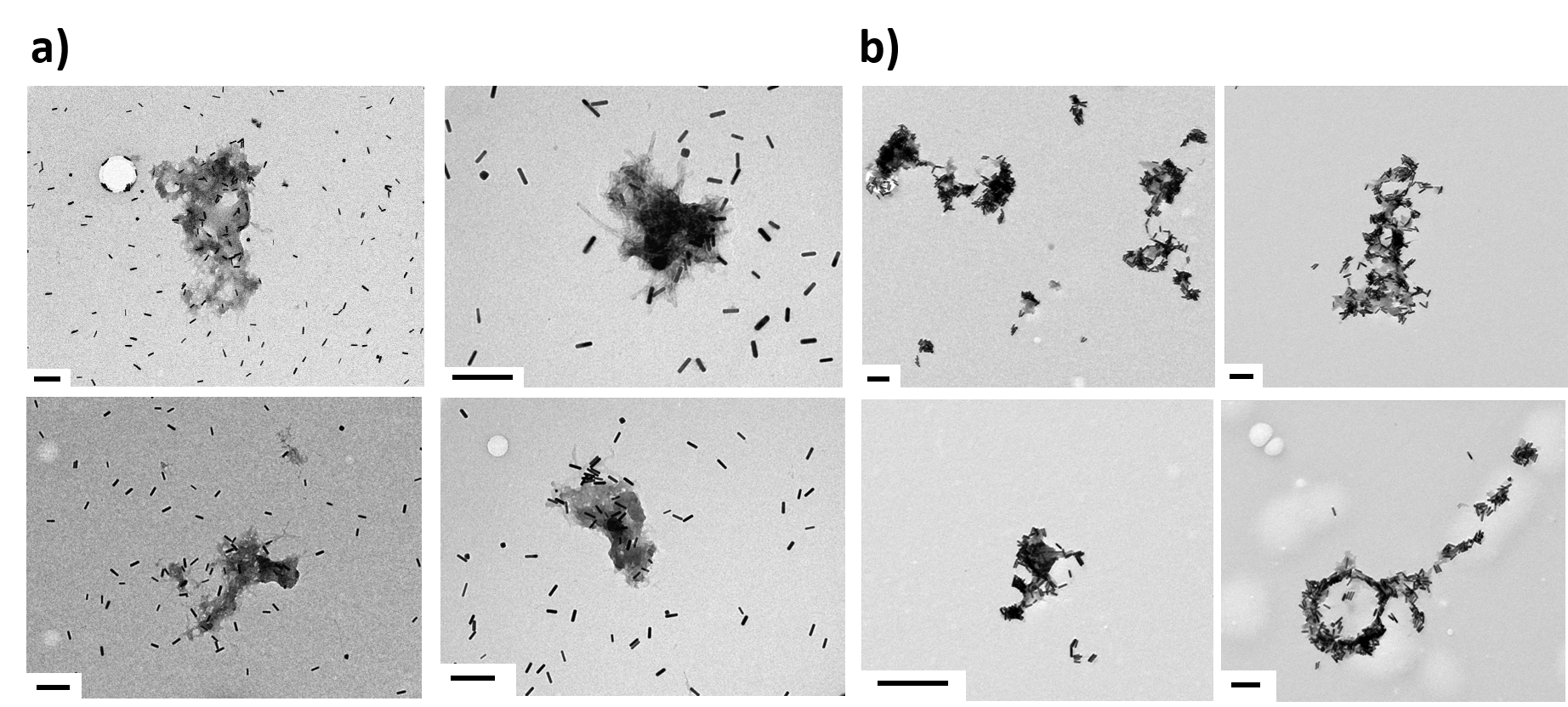

Supplement: S6 Fig — a) PMA-GNRs and b) Abs-GNRs after incubation with Aβ seeds overnight. Scale bars correspond to 200 nm. (TIF) [file pone.0259608.s009.tif]

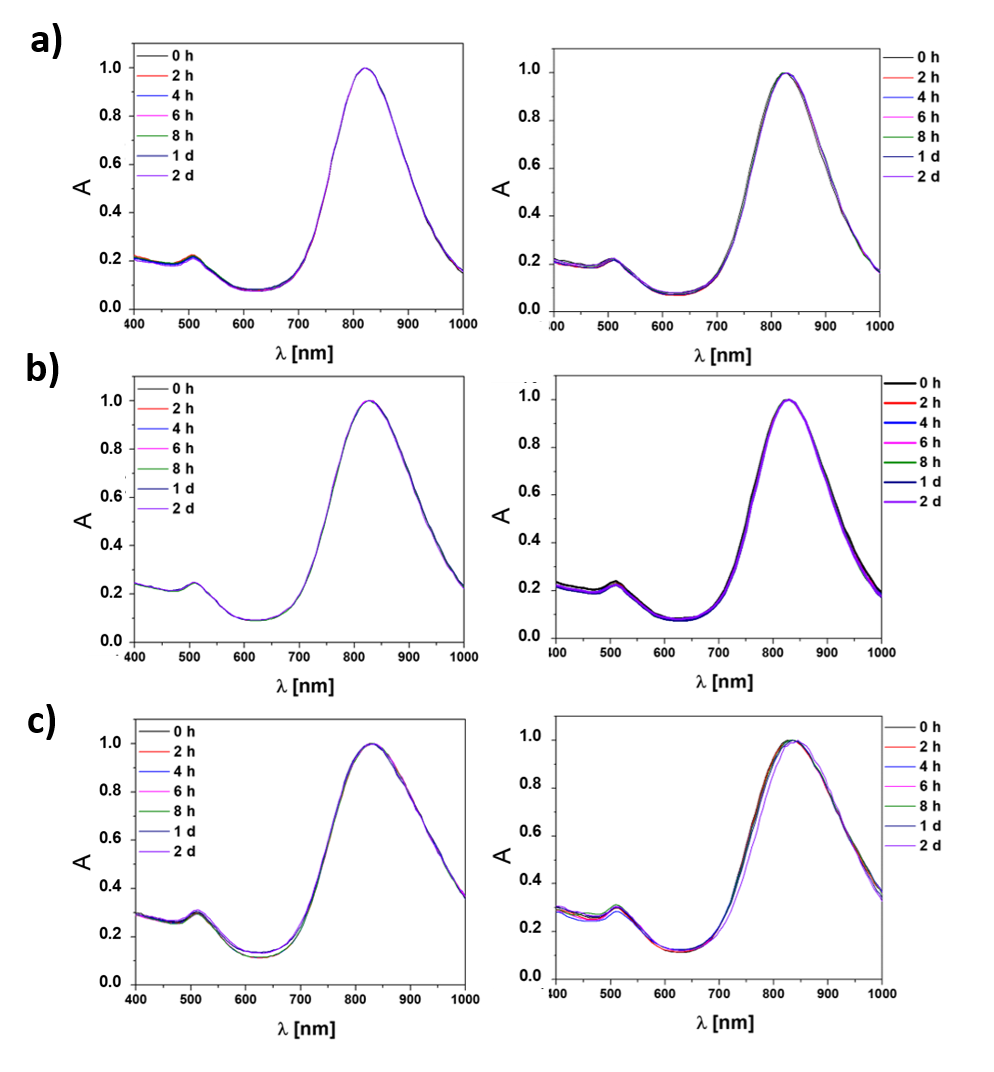

Supplement: S7 Fig — a) PMA- GNRs, b) Abs- GNRs, and c) seeds-Abs-GNRs in water (left) and cell media (right) at different point of times (up to 2 days). (TIF) [file pone.0259608.s010.tif]

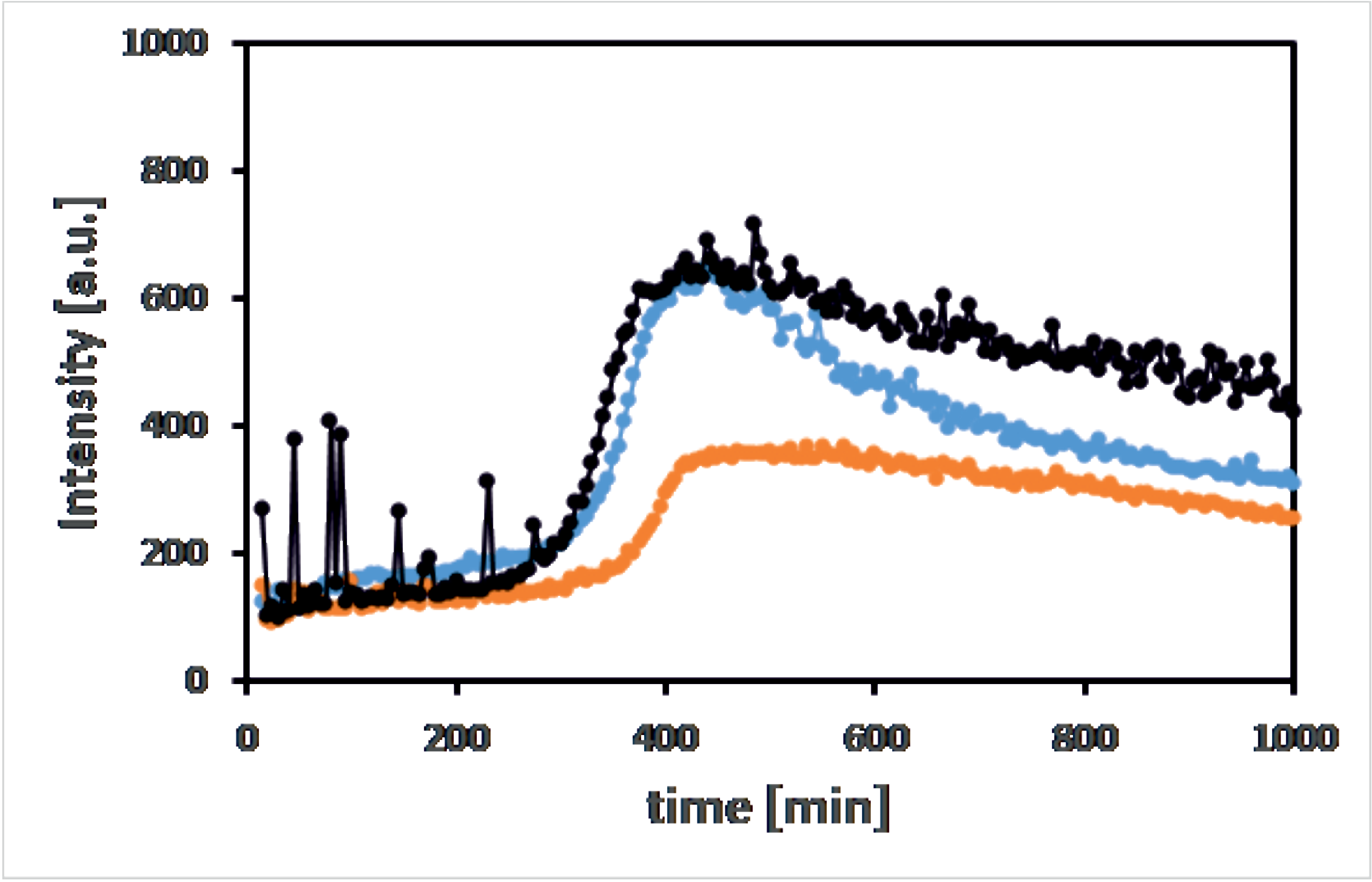

Supplement: S8 Fig — ThT fluorescence of 10 μM Aβ40 in PBS (pH 7.4, 37°C) alone (black), and in the presence of PMA-GNRs (light blue) or Abs-GNRs (orange). (TIF) [file pone.0259608.s011.tif]
