# Supplementary material for: Development of amyloid beta gold nanorod aggregates as optoacoustic probes
Source: PLoS One. 2022 Mar 25;17(3):e0259608. doi: 10.1371/journal.pone.0259608 (PMC8956182; doi:10.1371/journal.pone.0259608)
Supplement: S1 Appendix — (DOCX) [file pone.0259608.s012.docx]

**Calculating the amount of PMA needed for phase transfer of NPs from organic solvent to aqueous medium**

To determine the amount of polymer which is needed for the coating of the NPs, a calculation based on the total NP surface to be coated was done following the calculations previously reported by Pellegrino et al.,^[[1]](#endnote-1)^ in which the total effective surface area of one nanoparticle (A_eff_) is calculated in agreement with its shape. Nanorodswere considered as cylinders to simplify the calculations. In this case, the effective diameter (d_eff_) value included the diameter of the Au core as determined by transmission electron micoscopy (TEM) (d_c_, cf. section 3) plus two times the assumed thickness of the surfactant shell (L_s_ = 1 nm). Once the surface of one NP is determined then, knowing the NPs concentration (c_NP_) and the volume of the NP solution to be coated (V_NP,sol_), the total area (A_tot_) of the NPs in the solution can be determined by the formula (S1):

| \| $A_{\mathrm{eff}}=2\pi\cdot\left( \frac{\left( d_{c}+ 2L_{s} \right)}{2} \right)\cdot{(L}_{c}+2L_{s})$ \| (Eq. S1) \| \| --- \| --- \| |  |
| --- | --- | --- | --- |

A_eff_ is the effective surface area of one NP including the organic surfactant layer, d_c_ is the diameter of the Au core and L_c_ is the length of Au core of the gold nanorodsas determined by TEM. L_s_ corresponds to the thickness of the surfactant layer and d_eff_is the effective diameter, equal to the NP core diameter determined by TEM (d_c_) plus two times the assumed thickness of the surfactant shell (L_s_ = 1 nm). The total effective surface area (A_tot_) of NPs thus is:

| $A_{\mathrm{tot}}=c_{\mathrm{NP}}\cdot V_{NP,sol}\cdot N_{A}\cdot A_{\mathrm{eff}}$ | (Eq. S2) |
| --- | --- |

c_NP_ is the NP [M] concentration of the NP solution used of the coating. V_NP,sol_ [L] refers to the volume of the NP solution, and N_A_ is the Avogadro constant. Then, the amount of polymer needed to be added to the NP solution can be determined by formula S3.

| $V_{P}=\frac{N_{P}}{c_{P}}=\frac{A_{\mathrm{tot}} \cdot R_{P/Area}}{N_{A} \cdot c_{P}}$ | (Eq. S3) |
| --- | --- |

N_P_ [mol] is the amount of polymer molecules (given in terms of polymer monomers) needed to coat all the NPs in solution. c_P_ [M] is the concentration of the polymer stock solution. V_P_ [L] is the volume of polymer stock solution which needs to be added to the NP solution. A_tot_ [nm^2^] is the total surface area of all the colloidal NPs in the solution and R_P/Area_ is the number of monomers to be added per nm^2^ of effective NP surface. N_A_ is the Avogadro constant.

**References**

1. Pellegrino, T.; Manna, L.; Kudera, S.; Liedl, T.; Koktysh, D.; Rogach, A. L.; Keller, S.; Rädler, J.; Natile, G.; Parak, W. J., Hydrophobic Nanocrystals Coated with an Amphiphilic Polymer Shell: A General Route to Water Soluble Nanocrystals. Nano Letters 2004, 4, (4), 703- 707. [↑](#endnote-ref-1)
